# The unseen risk of Ozempic: NAION and vision damage

**DOI:** 10.1097/MS9.0000000000004149

**Published:** 2025-10-20

**Authors:** Noor Ul Ain Saleem, Mishaim Khan, Christian Tague, Aymar Akilimali

**Affiliations:** a FMH College of Medicine and Dentistry, Lahore, Pakistan; b Department of Research, Medical Research Circle (MedReC), Goma, DR Congo

**Keywords:** NAION, Ozempic, unseen risk, vision damage

## Abstract

Ozempic (semaglutide) was first authorized to treat type 2 diabetes, but because of its appetite-suppressing properties, it has become popular off-label for weight loss. According to clinical investigations, GLP-1 significantly decreases blood sugar and helps people lose weight. Approximately 15 million persons in the United States are currently taking GLP-1 drugs. Off-label use is becoming more common, which raises worries about potential health hazards and significant side effects such as non-arteritic anterior ischemic optic neuropathy (NAION). According to a study, 6.7% of people who were overweight and 8.9% of those with diabetes who took semaglutide had NAION. The FDA issued a warning regarding counterfeit and compounded products while approving semaglutide for weight loss under the Wegovy^®^ brand. Unsupervised usage and problems with diabetes accessibility raise ethical questions.

Ozempic (semaglutide), a glucagon-like peptide-1 receptor agonist, was initially approved by the US Food and Drug Administration to treat type 2 diabetes, a chronic condition characterized by the body’s inability to regulate blood sugar levels effectively^[1,2]^. Ozempic is becoming increasingly well-liked because of its proven effectiveness in clinical trials, which have shown that it can significantly reduce blood sugar levels and help people with type 2 diabetes lose weight. About 1 in 8 US adults have taken a GLP-1 medication at some point in their lives, and half of them, roughly 6% of adults, or more than 15 million people are currently on a prescription, according to recent KFF survey data^[3]^. An analysis of semaglutide use in Denmark between 2018 and 2023 revealed that the percentage of new users without a type 2 diabetes diagnosis rose from 1% in 2018 to 33% in 2022, suggesting a rise in off-label use for weight control^[4]^. In recent years, Ozempic’s sales have increased significantly. Ozempic’s sales in 2022 were $8.5 billion, with the United States accounting for 65% of that total. Sales for Ozempic are predicted to rise even more, hitting $12.5 billion in 2023 and $17 billion in 2029. This upward trend indicates growing demand for the drug, particularly in the US, underscoring Ozempic’s increasing prominence in the pharmaceutical industry^[5,6]^.

Healthcare professionals and researchers have reported a concerning correlation between the use of Ozempic and the development of non-arteritic anterior ischemic optic neuropathy (NAION), a rare but potentially serious eye condition that, if untreated, may result in irreversible vision loss with no proven permanent treatment actually exists^[6]^. Semaglutides cause NAION by vascular dysregulation at the optic nerve head, activation of GLP-1 receptors in retinal ganglion cells, and fast glycemic or weight changes that influence perfusion. Patients with packed optic discs or vascular risk factors may be more vulnerable^[7]^. Many experts are raising concerns about the potential risks that patients may face from these medications. These include side effects like muscle loss, severe side effects like pancreatitis, and kidney and gallbladder issues. In addition, there is a chance that these drugs could lead to eating disorders, and fake versions of these drugs can be dangerous^[8]^.

NAION is a complex condition that usually affects people older than 50. It is especially common among those with health issues like diabetes, high blood pressure, or obstructive sleep apnea. It also occurs in individuals who have “crowded” optic discs. A retrospective cohort study involving 16 827 neuro-ophthalmology patients found that over 36 months, the cumulative incidence of NAION was 8.9% in diabetic patients taking semaglutide compared to 1.8% in those using other antidiabetic agents (HR: 4.28, 95% CI: 1.62–11.29). In overweight or obese patients, the rates were 6.7% versus 0.8% (HR: 7.64, 95% CI: 2.21–26.36)^[6]^. However, these numbers represent a 3-year cumulative event rate in a specialized clinical group, not the overall annual incidence in the general population, which stays around 2 to 10 cases per 100 000 each year^[9]^. However, the study’s observational design and the specific nature of the patient group introduce potential issues. The findings show a link but do not prove causation. Therefore, while semaglutide may be linked to a higher risk of NAION, the absolute risk is still low, and there is need of more extensive studies with generalizable results.

FDA has now approved semaglutide for weight loss under the brand name Wegovy^®^ (2.4 mg weekly) and is being used off-label more and more via Ozempic^®^ and Rybelsus^®^ because insurance coverage varies^[10]^. Healthcare providers and regulatory bodies have expressed concern about the drug’s off-label use for weight loss, particularly among people who do not have diabetes. This off-label use has raised ethical and medical concerns, particularly about accessibility for diabetics and the potential side effects of unsupervised use. According to the FDA, patients are advised to obtain semaglutide only from registered pharmacies and licensed healthcare providers to minimize health risks.

As we navigate this complex terrain, we must have informed discussions about responsible prescribing practices and patient education to successfully lower these risks and ensure that patients understand the importance of taking medications as prescribed rather than caving into trends influenced by anecdotal evidence and social media. While routine eye screening is not recommended at this time, clinicians should be alert to at-risk patients who report visual symptoms. Furthermore, transparent communication between patients and medical staff can lead to more customized treatment plans, improving the overall effectiveness of treatments and reducing the risks associated with medications like Ozempic. This collaborative approach not only fosters trust but also empowers patients to actively monitor off-label use and improve healthcare management, ensuring that any negative consequences are addressed and managed effectively.

## Data Availability

Not applicable.
